# Do audio-visual motion cues promote segregation of auditory streams?

**DOI:** 10.3389/fnins.2014.00064

**Published:** 2014-04-07

**Authors:** Lidia Shestopalova, Tamás M. Bőhm, Alexandra Bendixen, Andreas G. Andreou, Julius Georgiou, Guillaume Garreau, Botond Hajdu, Susan L. Denham, István Winkler

**Affiliations:** ^1^Pavlov Institute of Physiology, Russian Academy of SciencesSt.-Petersburg, Russia; ^2^Research Centre for Natural Sciences, Institute of Cognitive Neuroscience and Psychology, Hungarian Academy of SciencesBudapest, Hungary; ^3^Department of Telecommunications and Media Informatics, Budapest University of Technology and EconomicsBudapest, Hungary; ^4^Auditory Psychophysiology Lab, Department of Psychology, Cluster of Excellence “Hearing4all”, European Medical School, Carl von Ossietzky University of OldenburgOldenburg, Germany; ^5^Department of Electrical and Computer Engineering, Johns Hopkins UniversityBaltimore, MD, USA; ^6^Department of Electrical and Computer Engineering, University of CyprusNicosia, Cyprus; ^7^School of Psychology, Cognition Institute, University of PlymouthPlymouth, UK; ^8^Department of Cognitive and Neuropsychology, Institute of Psychology, University of SzegedSzeged, Hungary

**Keywords:** auditory stream segregation, bistable perception, auditory motion, audio-visual integration

## Abstract

An audio-visual experiment using moving sound sources was designed to investigate whether the analysis of auditory scenes is modulated by synchronous presentation of visual information. Listeners were presented with an alternating sequence of two pure tones delivered by two separate sound sources. In different conditions, the two sound sources were either stationary or moving on random trajectories around the listener. Both the sounds and the movement trajectories were derived from recordings in which two humans were moving with loudspeakers attached to their heads. Visualized movement trajectories modeled by a computer animation were presented together with the sounds. In the main experiment, behavioral reports on sound organization were collected from young healthy volunteers. The proportion and stability of the different sound organizations were compared between the conditions in which the visualized trajectories matched the movement of the sound sources and when the two were independent of each other. The results corroborate earlier findings that separation of sound sources in space promotes segregation. However, no additional effect of auditory movement *per se* on the perceptual organization of sounds was obtained. Surprisingly, the presentation of movement-congruent visual cues did not strengthen the effects of spatial separation on segregating auditory streams. Our findings are consistent with the view that bistability in the auditory modality can occur independently from other modalities.

## Introduction

In natural environments, our senses provide us with an abundance of incoming information, which must be analyzed and segregated in order to form veridical representations of perceptual objects. In particular, the auditory system needs to separate the information relating to concurrently active sound sources to construct a consistent and stable interpretation of the acoustic environment. The perceptual task of analyzing a sound mixture has been termed “auditory scene analysis” by Bregman (1990). In everyday situations, perception is usually multimodal; often the currently interesting sound source is also in the focus of our visual attention or can be brought into it at will. A number of studies have provided evidence that auditory perception is enhanced by integration of information from multiple sensory modalities (e.g., Sumby and Pollack, 1954; Grant and Seitz, 2000; Eramudugolla et al., 2011; for a review, see Recanzone, 2009). However, much less is known about whether and how visual cues can support sequential auditory scene analysis. Therefore, in the current study we tested the effects of congruent visual spatial cues on the segregation of two series of interleaved sounds generated by the same or two different stationary or moving sound sources.

The process of organizing sounds into coherent sequences termed “auditory streams” has been extensively studied with the help of the auditory streaming paradigm in which listeners hear a repeating triplet composed of two kinds of sounds (“ABA_ABA_”) which differ from each other in some acoustic feature (for reviews, see Moore and Gockel, 2002; Cusack et al., 2004; Snyder and Alain, 2007; Winkler et al., 2009, 2012). This stimulus configuration is most commonly perceived either as a single coherent sequence with a galloping rhythm (termed the “integrated” percept) or as two concurrent streams, one consisting of the A and the other of the B sounds (“segregated” percept; Van Noorden, 1975). When listeners are presented with long unchanging sequences of this type, they report spontaneous perceptual switches between these alternative sound organizations (Gutschalk et al., 2005; Denham and Winkler, 2006; Pressnitzer and Hupé, 2006; Kondo and Kashino, 2009; Denham et al., 2010; Hill et al., 2012). These perceptual switches occur even when the stimulus configuration strongly promotes one alternative organization over another (Denham et al., 2013). Although somewhat less commonly used, alternating (ABAB_) sequences are also typically perceived in terms of one integrated (alternating) or two segregated (A and B, separately) streams (e.g., Yabe et al., 2001; Shinozaki et al., 2003) and exposure to long alternating sequences results in perceptual bistability (perceptual bistability (Bőhm et al., 2013; Szalárdy et al., 2014).

Empirical evidence consistently shows that separation in various auditory features between the A and B sounds (such as frequency, pitch, timbre, loudness, amplitude modulation and spatial location; e.g., Vliegen and Oxenham, 1999; Grimault et al., 2002; Roberts et al., 2002; Szalárdy et al., 2013; for a review, see Moore and Gockel, 2002) acts as the main cue for auditory stream segregation. The larger the acoustic separation between the A and B sounds and the faster the presentation rate, the more likely that the ABA_ sequence is perceived in terms of two separate sound streams. Although separation in sound source location is regarded as a weak cue for auditory stream segregation (e.g., Culling and Summerfield, 1995; for a review, see Bregman, 1990), when combined with separation in tone frequency, it was shown to increase the probability of listeners reporting the segregated percept (Denham et al., 2010; Szalárdy et al., 2013). These effects, based on acoustic separation (perceptual dissimilarity) between the feature values of the A and B sounds, reflect the Gestalt principle of similarity.

Other Gestalt principles have also been shown to contribute to auditory scene analysis (e.g., “closure,” see Bendixen et al., 2010). The Gestalt principle of common fate refers to similarity in perceptual trajectories, rather than in single feature values. One example is the various acoustic transformations resulting from the movement of sound sources. Bőhm et al. (2013) tested whether the potential cues resulting from auditory motion can also help to segregate two interleaved tone sequences. They varied the motion pattern (stationary location vs. two different kinds of trajectories) and the dynamics of spatial separation (standing/moving side by side or standing apart/moving on separate trajectories) of the two sound sources. Although the results showed a clear effect of spatial separation in general, auditory motion *per se* did not exert a significant influence on the proportions of time in which the segregated and integrated organizations were perceived by the listeners. Following up on these findings, we designed the present experiment to investigate whether the auditory spatial and motion cues would become more effective in supporting stream segregation when presented together with congruent visual cues.

The ability of the human brain to link information from different modalities to the same object is termed multisensory integration. Binding information from different modalities is assumed to help to reduce noise within the perceptual system (see Stein et al., 2004; Koelewijn et al., 2010). A large number of crossmodal spatial audiovisual studies have focused on the effects of presenting a congruent or conflicting unimodal cue in one modality on the detection or discrimination of targets presented in a different modality (for reviews, see Spence et al., 2004; Wright and Ward, 2008). Whereas the results of earlier cross-modal studies showed asymmetries between the effects of visual information on auditory perception and vice versa, the currently prevailing view is that crossmodal exogenous spatial cuing effects can be demonstrated for all possible combinations of successively presented auditory and visual stimuli, although the magnitude of the spatial cuing effects induced by the crossmodal cues may be different (Spence, 2010). Further, whereas some studies concluded that multisensory cues are no more effective than unimodal cues in capturing spatial attention (Ward, 1994; Spence and Driver, 2000; Vroomen et al., 2001; Santangelo et al., 2006, 2008; Van der Burg et al., 2008), the results of some more recent studies have overturned this conclusion by showing that multisensory cues do capture spatial attention more effectively than unisensory cues, at least under conditions of high perceptual load (i.e., when participants are simultaneously engaged in a concurrent perceptually demanding task; for reviews, see Spence and Santangelo, 2009; Spence, 2010) and when the unisensory components of the multisensory signals are presented from more-or-less the same spatial location (Ho et al., 2007, 2009).

Although most research on multisensory interaction focused on the integration of static objects, there has been a recent growth of interest in multisensory interactions specifically influencing the perception of motion. A number of investigations employing apparent motion have demonstrated the existence of robust interactions between sensory modalities during the extraction of motion information (for a review, see Soto-Faraco and Väljamäe, 2012). In particular, studies of the effect of directional audiovisual congruency (cross-modal dynamic capture) demonstrated that sound motion discrimination performance can be substantially enhanced when a concurrent visual motion stream is synchronously presented from the same direction as the sounds, and eliminated when the visual and auditory signals are desynchronized in time. Further, the magnitude of the dynamic capture effect far exceeded what could have been expected on the basis of simple static capture, suggesting that motion-related cross-modal interactions are probably based on motion information *per se*, rather than on other features, such as the location and timing of the stimuli (Soto-Faraco et al., 2003, 2004).

Binding processes within and across sensory modalities have also been studied using multistable stimuli. There is substantial evidence that multistable effects in one modality can be modified by stimuli in another modality (Schwartz et al., 2012). It has been shown that sounds congruent with one or the other image may affect perceptual dominance proportions in binocular rivalry, but only when the visual stimulus is consciously perceived (Kang and Blake, 2005; Conrad et al., 2010). The visual influence on auditory perception in speech was examined using a speech stimulus that generated bistable perception and a dynamic version of the Rubin vase illusion (Munhall et al., 2009). The results indicated that visual influences on auditory speech processing, at least for the McGurk illusion, necessitate conscious perception of the visual speech cues, thus supporting the hypothesis that multisensory speech integration is not completed at an early processing stage. Van Ee et al. (2009) have shown that attentional control over the perceptual organization of an ambiguous auditory stimulus can be markedly aided by matching visual stimuli. They argued that the audiovisual binding that served awareness is not fully automatic: stimuli in one modality only influence the perception of an ambiguous stimulus configuration in the other modality when one attends to the stimulus presented in the first modality (Van Ee et al., 2009).

In the current study, we investigated whether congruent spatial visual information affects the segregation of two interleaved sounds sequences emitted by one or two stationary or moving sound sources. We hypothesized that congruent unambiguous visual spatial information would reduce the ambiguity of the auditory scene and thus bias the emerging auditory perceptual organization. That is, when participants are presented with sounds originating from one or two sound sources, congruent as compared to incongruent visual spatial information was expected to increase the proportion of time in which perception was veridical.

## Methods

### Participants

Twenty-five young adults (10 females; 23 right-handed; 18–26 years, mean age 21.6 years) took part in the experiment for modest financial compensation. Participants were screened in advance for (1) normal hearing (thresholds below 25 dB above hearing level, measured at 250, 500, 1000, 2000, and 4000 Hz, and a maximum threshold difference between the two ears of 10 dB at 250 Hz, 5 dB at 500 and 1000 Hz, 10 dB at 2000 and 4000 Hz were required) and for (2) the ability to detect synchrony between fluctuations in auditory and visual stimuli (see in the *Procedure* section). All participants had normal or corrected-to-normal eyesight. Because our measure of the effects of the stimulus manipulations requires that listeners experience perceptual switches, the data provided by four participants were discarded from the analysis, as they did not experience/report perceptual switches in five or more stimulus conditions. (All other listeners experienced/reported perceptual switches in all but two or fewer stimulus conditions.) Thus the analyses are conducted on data from 21 listeners (10 females, 19 right-handed, 18–26 years, mean age 21.5 years). Participants gave written informed consent after the aims and procedures of the experiment were explained to them. The study was approved by the Ethical Committee of the Institute of Cognitive Neuroscience and Psychology, Research Center for Natural Sciences, Hungarian Academy of Sciences.

### Apparatus and stimuli

#### Audio recordings

The auditory stimuli were based on audio recordings made on the stage of the Ceremony Hall of the University of Cyprus. Preparatory tests showed no significant reverberations in this area. Alternating sequences of two 100 ms long (including onset and offset ramps of 10 ms long raised cosines) pure tones of equal amplitude were recorded with 25 ms inter-stimulus interval. One tone had a frequency of 400 Hz (subsequently denoted “low”), the other was three semitones higher at 475.7 Hz (subsequently denoted “high”). The tone sequences were played by Anthony Gallo Acoustics A'Diva Ti compact speakers mounted on construction helmets worn and carried around by two experimenters (TMB and GG). The speaker drivers were facing upwards to provide equal sound emission in horizontal directions. The sound signals were generated on an IBM PC and transmitted to the speakers by FM radio units. Sounds were recorded with a Head Acoustics HSU III.2 head microphones mounted within the artificial head placed at the center of the stage, and digitized with a National Instruments 4462 data acquisition card of an IBM PC at a sampling rate of 96 kHz and 24 bits resolution. In order to reduce noise inherent in on-site sound recordings, the audio signals were post-processed by removing the DC offset and bandpass filtering in the 350–526 Hz range with a third-order Butterworth filter.

In order to create 4-min-long stimulus blocks for each experimental condition, appropriate segments were selected from the audio recordings and extended to exactly 4 min by looping. We chose the longest possible segment of the signal that had good recording quality throughout and could be looped. We avoided introducing discontinuities into the spatial trajectories by selecting sections in which the initial and final estimated interaural time differences and their first derivatives roughly matched (separately for the high and the low tones). The endpoints of the sections were always placed in the middle of the silent interval before a low tone. While looping the audio segments to block length, a cross-fading procedure was applied in the 1 ms vicinity of the concatenation points to prevent audible clicks. The length of the segments chosen ranged between 20 and 64 s. Finally, the signals were re-sampled at 192 kHz and their intensity was normalized across the segments. The alternating (ABAB) tone sequence was chosen for the recordings because it provided more potential section endpoints than the repetitive triplet pattern (ABA_) typically used in the auditory streaming paradigm (Van Noorden, 1975). In a pilot study, we found that using identical stimulus parameters (pitch separation and presentation rate), the ABAB and ABA_ patterns produce similar distributions of the reported percepts.

#### Visual stimuli

The spatial positions of the helmet-loudspeakers were recorded by means of a Microsoft Kinect device connected to a PC and interfaced to Matlab through the Open Natural Interaction toolbox. The helmets were painted red and blue to facilitate their tracking in the video data. The Kinect's depth sensor provided a three dimensional map of its field-of-view while a color video was recorded by its camera sensor. The trajectory data was smoothed by median filtering and moving averaging, and re-sampled to 20 frames-per-second. Visual stimuli for the experiment were then created from the movement trajectories using Unity 3D computer animation software. The sound-emitting moving objects were represented by two colored balls (one red, the other blue) moving along the movement trajectories in a three dimensional room-like space. The videos had a refresh rate of 40 Hz which provided frame durations of 25 ms. Thus each sound was covered exactly by four frames in the video and the inter-stimulus interval by a single frame, which allowed the colored balls to pulsate in full synchrony with the presentation of the corresponding tones. Pulsation was created by modulating the size of the visual objects with a size ratio of 10% between the “sound-on” and “sound-off” states. The audio and video recordings were synchronized by storing timestamps for the beginning of each sound recording and for each video frame captured by the Kinect.

#### Conditions

**Figure 1:**
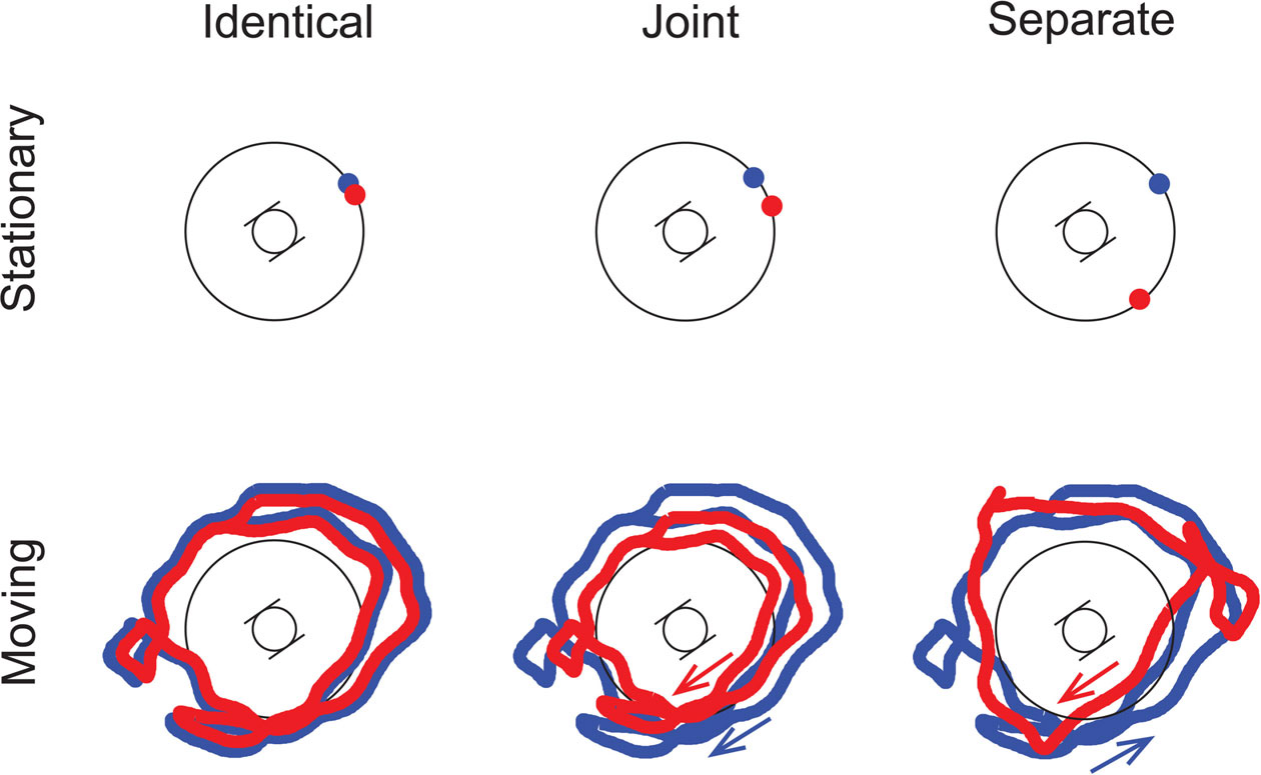
**Schematic illustration of the spatial trajectories of the sound sources.** The artificial head was placed at the center of the stage, and listeners perceived the stimuli as if sitting in this position. The two trajectories are drawn in red and blue. For *Joint* and *Separate* motion, arrows indicate the direction of movement for each trajectory. Note that the trajectories are illustrations only; they do not depict the actual recordings. (Adapted by permission from Figure 1, Bőhm et al., 2013.)

The two experimenters, each wearing a helmet with the loudspeaker on top, performed a number of different scenarios during the recording session (cf. Figure 1). Each experimental condition was based on the recording of a separate scenario. The two kinds of sound Motion scenarios tested were *Stationary* (based on scenarios with both experimenters standing still) and *Moving* (the experimenters were walking on a random trajectory around the artificial head). The Co-location of the two sound sources could either be characterized by *Identical* (based on recordings with one experimenter standing or moving, his helmet-loudspeaker delivering the full alternating sequence), *Joint* (the two experimenters stood or moved together hand-in-hand, i.e., the distance between their trajectories being roughly constant, with one loudspeaker delivering the high, the other the low tones), and *Separate* trajectories (when the two experimenters were standing or moving separately, again the two loudspeakers delivering different tones) (see Figure 1). The cross-modal Congruency of the visual and auditory stimuli was represented by the *Congruent* (the visualized movement matched the auditory movement of the objects) and the *Incongruent* conditions (the visualized movement was independent of the auditory movement). The auditory stimulation in the *Congruent* and *Incongruent* conditions was the same. The conditions were fully crossed, leading to 12 stimulus conditions in total. Samples for the *Congruent*/*Moving*/*Separate* and *Incongruent*/*Moving*/*Joint* conditions can be found at http://figshare.com/articles/Stimulus_Congruent_Moving_Separate/961766 and http://figshare.com/articles/Stimulus_Incongruent_Moving_Joint/961765.

The visual stimuli were made incongruent by applying four transformations to the spatial trajectories of the two visual objects: (1) the trajectories of the two objects were exchanged (i.e., the recorded trajectory of the “blue” object served as the basis of the incongruent trajectory of the “red” object and *vice versa*), (2) the trajectories were inverted in time, (3) the trajectories were mirrored producing a left-right flip (as rendered on the screen), and (4) the trajectories were rotated in the horizontal plane around the view-point of the observer by a random amount. The amount of rotation was drawn from a uniform distribution independently for each listener and condition, so that (a) it was between 30 and 330° and (b) the rotated trajectory fell into the field-of-view of the animation for the *Stationary* conditions. The same random amount of rotation was applied to the trajectories of both visual objects, except for the *Moving/Separate* conditions. In the latter case, the amounts of rotation of the two trajectories were independently drawn. As a result, the incongruent trajectories had the same statistical properties as the congruent ones (e.g., the distribution of velocities was the same), and the type of visual motion was compatible with that delivered by the audio (e.g., the two objects moved randomly, independently from each other, both in the video and the audio). However, the *Incongruent* videos differed from the *Congruent* ones sufficiently to prevent binding of the auditory and visual stimuli (i.e., the objects could be seen definitely elsewhere from where the sound came from).

### Procedure

Participants were preselected on the basis of the results of a synchrony test designed to check whether they were able to bind auditory and visual stimuli into a single perceptual object. To this end, before enrolling a participant on the experiment, they were presented with a demonstration of the stimuli that consisted of 7 examples of 20 s duration each. For each example, the participant was asked to report whether he/she recognized the visual objects as sound sources. If not, the example was repeated a few times. Only participants who reported experiencing the sounds as originating from the visual objects in the congruent examples were entered into the next phase of the experiment. In the first three examples, a single stationary red ball (identical to the one created for the main experiment) was shown at the center of the screen. In the first example it was pulsating at a rate of 1 Hz, in full synchrony with the tone sequence. Next an “asynchronous” example was presented which employed the same visual and auditory stimuli but the sound sequence was delivered with a 500 ms delay. In all other examples the sound sequences were pulsating synchronously with the visual stimuli. In the third example the pulsation rate of the visual object and the sound sequence was increased to 2 Hz. After these examples, two visual objects (a red ball in the center and a blue ball on the left side of the screen) were displayed, first with a pulsation rate of 2.67 Hz and then with 8 Hz, the latter being equal to the presentation rate used in the main experiment. For these demonstrations, the trajectories of the visual objects and the sound sequences were taken from the *Stationary*/*Separate* condition. To create the examples with lower pulsation rate, the necessary number of sound bursts was periodically muted, resulting in a sound sequence that could be experienced as pulsating synchronously with the visual stimuli. In the last two examples, the *Moving*/*Separate* condition was taken as the basis for creating both the auditory and the visual stimuli. Two visual objects were moving separately and they pulsated at a rate of 2.67 Hz in the first example and at 8 Hz in the second one.

After these demonstrations, synchrony tests containing 2–5 four-trial blocks were conducted. On each trial the participant received an audiovisual stimulus of 20 s duration representing the *Stationary*/*Separate* condition (with the red and the blue ball placed at the center and on the left side of the screen, respectively). The sounds were presented at the rate of 8 Hz and on half of the trials, the visual object pulsated in full synchrony with the tone sequence. On the other half of the trials, the visual pulsation rate differed from the tone presentation rate by 30% (on half of these trials it was higher, on the other half it was lower). The test ended when the participant correctly judged the synchrony of at least 7 trials within 2 consecutive four-trial blocks, or the maximum number of trials (20 trials; 5 blocks) was reached. Participants who reached the criterion continued with the second test. The second test only differed from the first in that the visual pulsation rate could differ from the tone presentation rate by only ±20%. Participants passed this test by correctly judging synchrony for at least 6 trials within 2 consecutive blocks of trials before the maximum number of trials (20) was reached. Participants were only enrolled in the study if they passed both tests. Those who did not pass were enrolled in a different experiment.

Each condition was administered in one 4-min long stimulus block during the experimental session. The order of the 12 conditions was randomized separately for each participant. There were short breaks lasting about 30 s between successive stimulus blocks and participants could choose to have a 5-min break after any block (or they had it at the 8th block, if they did not ask for it before). The stimuli were played by an IBM PC with an Audiotrak Prodigy HD2 sound card. Sounds were amplified by a custom-made mixer-amplifier and delivered by Etymotic Research ER-2 insert earphones. The insert earphones provided at least 30 dB external noise attenuation and made sure that participants heard the sounds as if standing where the artificial head was located at the time when the sounds were recorded. Thus the binaural location cues related to head-related transfer functions were adequately reproduced, while the monaural cues (e.g., those associated with pinna) were shown not to contribute greatly to stream segregation in a similar sound configuration (Bőhm et al., 2013). The sound level was set to 50 dB above the hearing threshold of the participant, as determined immediately before the experiment in a simplified staircase measurement using a sequence alternating the two tones (400 and 475.7 Hz). The visual stimuli were presented with an NVIDIA GeForce 9500 GT video card at a resolution of 1280*720 pixels and delivered on a Samsung LE40C530F1W LCD TV with a screen diameter of 101 cm. The participant was seated 35 cm in front of the TV screen resulting in a 103° wide viewing angle of the scene. The participant's head was supported by a chin holder to avoid head movements during the stimulus blocks. The colored balls came off-screen when their location exceeded the viewing angle of the TV screen. (This never happened in the Stationary conditions.) Given that the random trajectories of the movement of the sources provided an approximately even distribution of the horizontal viewing angle, the amount of time the balls spent off-screen was proportional to the viewing angle and it approximately corresponded to how a viewer with fixed head direction would have seen the sources in a real-life scene.

Prior to the main experiment, participants received training for the types of patterns they were asked to report using the keys. The “integrated” and “segregated” percepts were explained and illustrated with sound examples to the participants before the experiment and the experimenter made sure they understood the task. Because there is no single prototype for the “both” percept, listeners received explanations but not sound examples for these patterns.

During the main experiment, participants were comfortably seated in an anechoic chamber and were instructed to continuously report the perceived sound organization throughout the entire stimulus block using two response keys, one key held in each hand. They were to keep one key depressed as long as they perceived both high and low tones as part of a single repeating pattern (termed the “integrated” percept). The other key was to be kept depressed as long as they heard tones of the same pitch forming separate repeating patterns (the “segregated” percept). Whenever they heard both types of patterns concurrently, they were to keep both keys depressed (the “both” percept). During those times when the participant did not perceive any repeating sound pattern, he/she was instructed to release both keys (the “neither” percept). The possibility of four choices, as opposed to forcing listeners to choose between reporting integrated or segregated percepts, has been introduced after we found that asking listeners to report their perception verbally in an unconstrained manner resulted in more than two alternatives (Denham et al., 2013; for an exploration of the perception of different patterns, see Denham et al., 2014). The instructions emphasized that the appropriate key combination was to be held as long as the participant perceived the corresponding sound pattern but to be changed immediately when the perceived pattern changed to a different category. Participants were informed that there was no correct or incorrect way to perceive any of the stimulus sequences; therefore they should not try to force to hear the sounds in one or another way. Rather, they should report what they actually hear. A description of the interpretation of the percepts reported by depressing both keys at the same time can be found in Denham et al. (2013). The assignment of the two response keys was counterbalanced across participants.

Besides judgments about the perception of the tone sequences, participants were also given a secondary task the motivation of which was to help binding between the corresponding audio and visual stimuli. The blue and red balls modeling the movement trajectories blinked 5–15 times (uniform distribution) during each 4-min stimulus block, so that one ball at a time became white for 100 ms before returning to its original color (blinks occurred equiprobably and in a random order on the two visual objects). The participant was instructed to count how many times the balls blinked (all blinks, irrespective of on which object they occurred). Following each stimulus block, the participant received feedback on the reported blink count.

### Data recording and analysis

The state of the two response keys was sampled at a 40 Hz rate, and the data were analyzed similarly to the procedure used in Denham et al. (2013). For each perceptual phase (i.e., the time interval between two consecutive perceptual switches), the logarithm of its duration in milliseconds and the reported percept was extracted. Perceptual phases shorter than 300 ms were excluded from the analysis as these presumably originate from inaccurate timing of button presses and releases, rather than from two separate perceptual switches quickly following each other (Moreno-Bote et al., 2010). Based on this data, for each participant, the proportions of the different percepts (i.e., the percent of time the listener experienced a given percept within the stimulus block) and mean log perceptual phase durations were calculated, separately for each perceptual organization (the “integrated,” “segregated,” and “both” percepts), and condition. The “neither” responses were not analyzed as they appeared in less than 1.4% of the stimulus block time in any of the conditions.

Repeated measures analyses of variance (Three-Way ANOVAs) were carried out separately for each of six variables (proportion and mean phase duration, separately for the “integrated,” the “segregated,” and the “both” percept) with the structure: Congruency [*Congruent* vs. *Incongruent*] × Motion [*Stationary* vs. *Moving*] × Co-location [*Identical* vs. *Joint* vs. *Separate*]. Where applicable, degrees of freedom were adjusted with the Greenhouse-Geisser correction factor (ε). These and the partial η^2^ effect sizes are reported for all significant effects. *Post-hoc* comparisons for the 3-level factor Co-location were performed using Tukey's HSD tests. Interaction effects between Co-location and Congruency on the proportion of the segregated and the integrated percepts were followed up by separately testing the effects of Co-location for the two levels of Congruency with repeated measures ANOVAs. All analyses were carried out at the 95% confidence level.

The level of performance in the secondary task was assessed by the blink count error (the difference between the reported and the actual blink count) established separately for each condition and listener. Negative values correspond to underestimation and positive values to overestimation of the number of blinks. Counting accuracy was estimated separately for each listener and condition by dividing the unsigned blink error value by the number of the blinks in the condition. The resulting values were analyzed by repeated measures analyses of variance (Three-Way ANOVAs) with the same factors as used for the main perceptual variables (Congruency [*Congruent* vs. *Incongruent*] × Motion [*Stationary* vs. *Moving*] × Co-location [*Identical* vs. *Joint* vs. *Separate*]).

## Results

**Figure 2:**
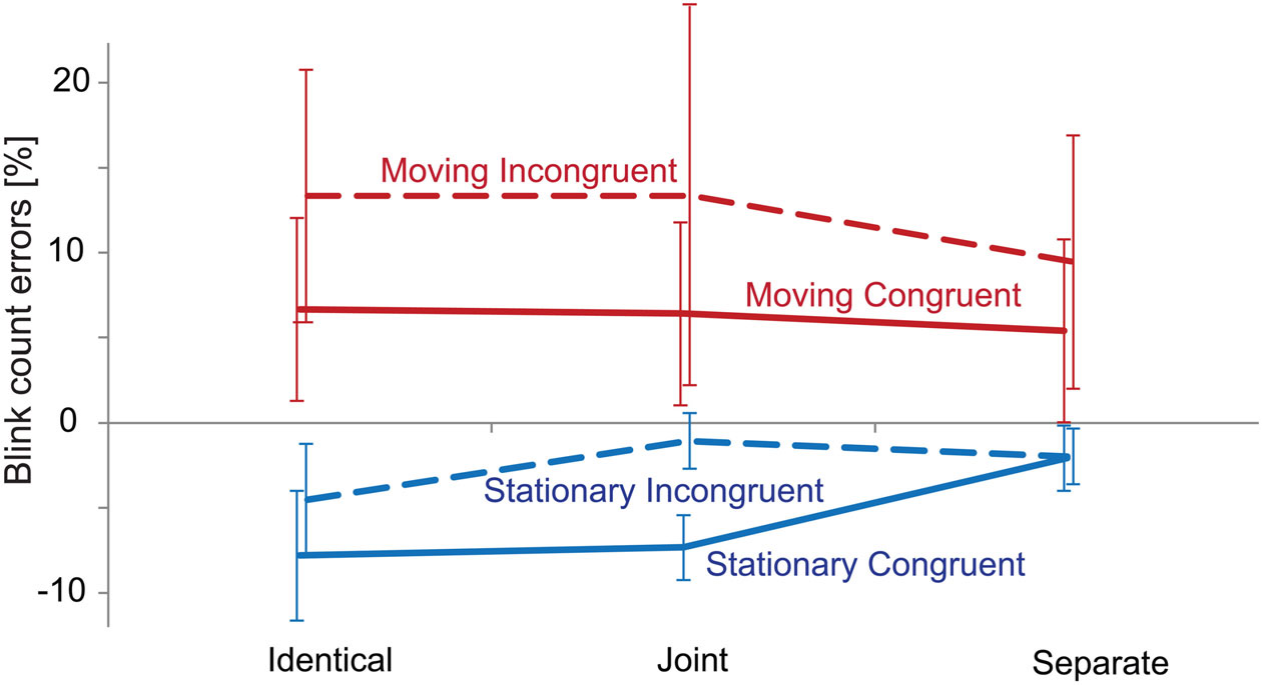
**Performance in the visual (blink counting) task.** Group-averaged (*N* = 21) percentage of blink count errors obtained in the *Identical, Joint* and *Separate* Co-locations, separately for the *Stationary* (blue) and *Moving* (red) × *Congruent* (continuous line) and *Incongruent* (dashed line) conditions. Negative values correspond to underestimation and positive values to overestimation of the number of blinks. Error bars show the standard error of means.

Figure 2 presents the group-average percentage of signed blink count errors (relative to the number of actual blinks), separately for each condition. The Three-Way ANOVA of the accuracy (unsigned) values yielded no significant main effect of Congruency. Motion had a significant main effect [*F*_(1, 20)_ = 7.46, *p* < 0.05, η^2^ = 0.272]. This was due to the lower number of errors made in the *Stationary* compared with the *Moving* conditions. No other main effects or interactions were significant.

**Figure 3:**
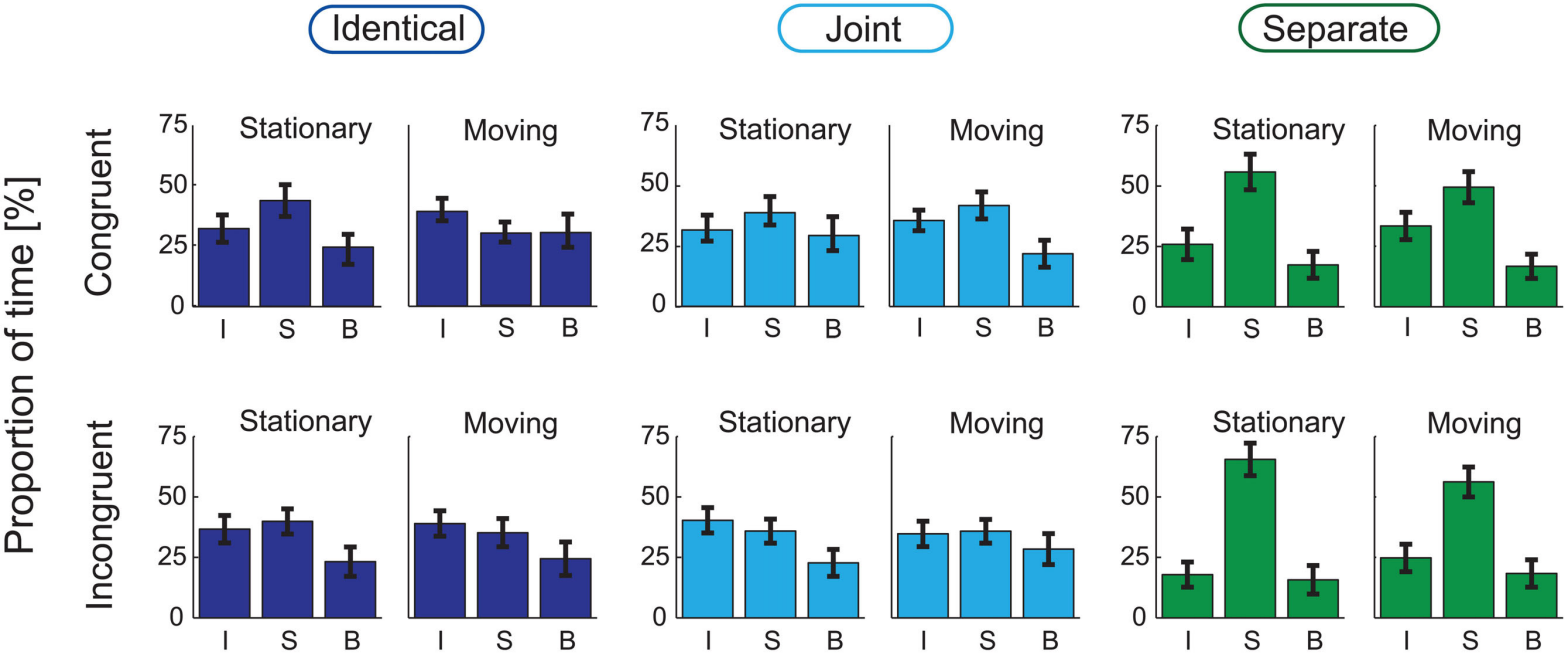
**Group-averaged (*N* = 21) percentages of time of the three response alternatives (I: “Integrated,” S: “Segregated,” B: “Both”) are shown for the *Identical* (left panel), *Joint* (middle), and *Separate* (right) Co-locations, separately for the *Stationary* (left, separately within the three panels) and *Moving* (right) conditions and for the *Congruent* (top row) and *Incongruent* (bottom) conditions.** Error bars show the standard errors of mean.

**Figure 4:**
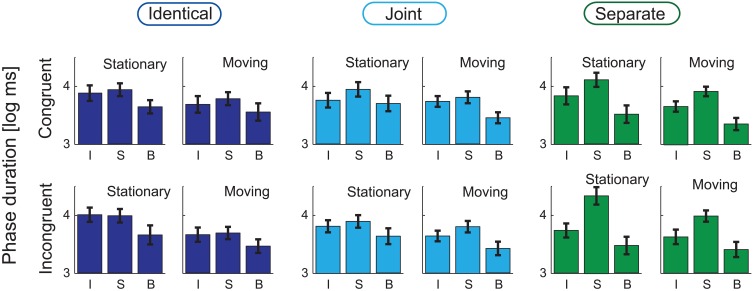
**Group-averaged (*N* = 21) average phase durations of the three response alternatives (I: “Integrated,” S: “Segregated,” B: “Both”) are shown for the *Identical* (left panel), *Joint* (middle), and *Separate* (right) Co-locations, separately for the *Stationary* (left, separately within the three panels) and *Moving* (right) conditions and for the *Congruent* (top row) and *Incongruent* (bottom) conditions.** Error bars show the standard errors of mean.

Figures 3, 4, respectively show the group-averaged proportions and mean phase durations of the perceptual alternatives reported by the listeners (“integrated,” “segregated,” and “both”) for all 12 stimulus conditions. Results of the ANOVA analyses are listed in Table 1.

**Table 1:** ***F* values yielded by the ANOVAs for the overall phase proportions and phase durations for the factors of Congruency (*Congruent* or *Incongruent*), Motion (*Stationary* or *Moving*), and Co-location (*Identical, Joint* or *Separate*)**.

ANOVA factors and interactions (*df*)	IntProp	SegProp	BothProp	IntDur	SegDur	BothDur
Congruency (1, 20)	0.11	0.45	0.31	0.07	1.48	0.38
Motion (1, 20)	0.81	1.45	0.71	19.27^***^	26.28^***^	12.87^**^
Co-location (2, 40)	7.69^**^	15.69^***^	2.81	1.72	12.79^***^	2.20
Congruency × motion (1, 20)	1.56	0.05	1.44	0.73	1.24	0.02
Congruency × co-location (2, 40)	3.70^*^	3.57^*^	0.46	0.65	2.46	0.20
Motion × co-location (2, 40)	1.28	2.55	0.61	1.39	2.60	0.82
Congruency × motion × co-location (2, 40)	0.38	0.81	1.51	0.68	0.64	0.62

The measures compared are denoted as following: IntProp, proportion of “integrated” phases; SegProp, proportion of “segregated” phases; BothProp, proportion of “both” phases; IntDur, average duration of all “integrated” phases; SegDur, average duration of all “segregated” phases; BothDur, average duration of all “both” phases. Degrees of freedom (df) are given in the row titles. Significance levels (p) are indicated by asterisks.

*** p < 0.001,

** p < 0.01,

* p < 0.05.

**Figure 5:**
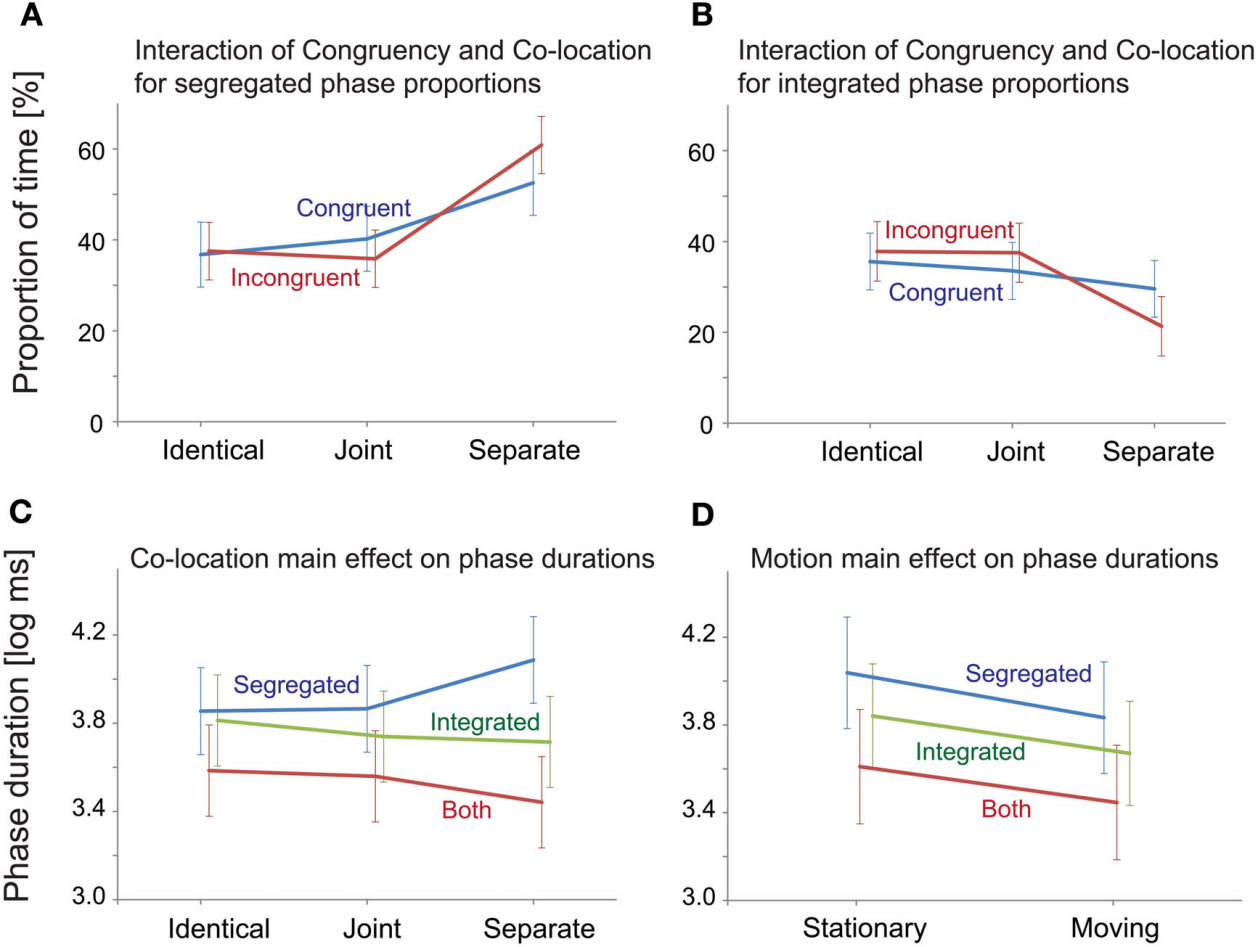
**Significant ANOVA effects for “Integrated,” “Segregated,” and “Both” response alternatives. (A)** Spatial separation increased the proportion of the “segregated” responses, this effect being larger with the *Incongruent* visual cues. **(B)** Spatial separation decreased the proportion of the “integrated” percepts, this effect being significant only with the *Incongruent* visual cues. **(C)** Spatial separation influenced the average phase durations for all percepts, this effect being significant only for the “segregated” phases. **(D)** Motion had a significant main effect on the log-mean phase durations of all percepts. In all panels, error bars show the standard errors of mean.

The analyses yielded no significant main effect of Congruency on any of the measured variables. Co-location had a significant main effect on the proportion of the “segregated” and “integrated” phases [*F*_(2, 40)_ = 15.69, *p* < 0.001, ε = 0.624, η^2^ = 0.440 for the “segregated” and *F*_(2, 40)_ = 7.69, *p* < 0.01, ε = 0.621, η^2^ = 0.278 for the “integrated” percept]. A significant interaction between Congruency and Co-location was obtained for the proportion of the “integrated” and “segregated” percepts [*F*_(2, 40)_ = 3.70, *p* < 0.05, ε = 0.954, η^2^ = 0.156 for the “integrated” and *F*_(2, 40)_ = 3.57, *p* < 0.05, ε = 0.973, η^2^ = 0.152 for the “segregated” percept]. In *post-hoc* ANOVAs the effects of Co-location were separately tested for the two levels of Congruency. In the *Congruent* condition, a significant effect of Co-location was obtained for the proportion of the “segregated” [*F*_(2, 40)_ = 6.52, *p* < 0.01, ε = 0.806, η^2^ = 0.246] but not for the “integrated” percept. In the *Incongruent* condition, Co-location had significant effects on the proportion of both the “segregated” and the “integrated” percept [*F*_(2, 40)_ = 18.32, *p* < 0.001, ε = 0.688, η^2^ = 0.478 and *F*_(2, 40)_ = 10.06, *p* < 0.01, ε = 0.698, η^2^ = 0.335, respectively]. Figures 5A,B shows that the interaction stems from the effects of Co-location being more pronounced in the *Incongruent* than in the *Congruent* condition: (1) the proportion of the “segregated” responses was higher for the *Separate* compared with the *Identical* and the *Joint* trajectories, this difference being larger with the *Incongruent* visual cues and (2) the proportion of the “integrated” percept was influenced by spatial separation of the sound sources only with the *Incongruent* visual cues: with *Separate* trajectories the proportion of the “integrated” percept was lower compared with the *Identical* and the *Joint* trajectories.

Figure 5C illustrates the main effects of Co-location on the log-mean phase duration of all percepts. This main effect was significant only for the “segregated” phases [*F*_(2, 40)_ = 12.79, *p* < 0.001, ε = 0.711, η^2^ = 0.390]. According to the *post-hoc* tests, the duration of the “segregated” percept was longer for the *Separate* compared with the *Identical* and the *Joint* trajectories (both *p* < 0.001).

Motion (see Figure 5D) had a significant main effect on the log-mean phase durations of all three percepts [*F*_(1, 20)_ = 26.28, *p* < 0.001, ε = 1, η^2^= 0.568 for the “segregated,” *F*_(1, 20)_ = 19.27, *p* < 0.001, ε = 1, η^2^= 0.491 for the “integrated,” and *F*_(1, 20)_ = 12.87, *p* < 0.01, ε = 1, η^2^= 0.392 for the “both” responses]. For all three percepts, this was due to shorter phase durations when the sound source was moving compared with that obtained for stationary sound sources.

## Discussion

We studied the effects of congruent visual spatial information on the perception of stationary and moving sound sources in a bistable auditory stimulus configuration, the auditory streaming paradigm. We expected that the audio-visual binding established during the training should result in participants experiencing more “veridical” percepts when the visual display was congruent with the spatial location/trajectory of the sound sources as compared to when they received incongruent visual information. “Veridical” entails the perception of two auditory streams when the original sound sources were separate and the perception of a single auditory stream when the original sound sources were identical. Specifically, we expected to find (1) increased proportions of segregated responses in *Congruent/Separate* as compared to *Incongruent/Separate* conditions and (2) increased proportions of integrated responses in *Congruent/Identical* as compared to *Incongruent/Identical* conditions. However, we found no clear effects of the congruency of visual information on auditory segregation or integration. Audio-visual congruency only appeared to significantly interact with the Co-location factor, the effect indicating stronger influence of spatial separation of the sound sources on perceptual organization in the *Incongruent* than in the *Congruent* conditions. Increased amounts of spatial separation between the sound sources resulted in a lower proportion of the “integrated” percept, the effect being significant only with incongruent but not with congruent visual cues. The proportion of the “segregated” percept significantly increased with increasing spatial separation between the sound sources both with congruent and incongruent visual cues.

One explanation for this somewhat unexpected result is that participants could have had difficulties in binding the auditory and visual cues of the abstract objects employed in the present experiment. However, participants were preselected on the basis of a synchrony test conducted after the training procedure checking whether they were able to bind the auditory and visual stimuli into a single multimodal perceptual object. Further, the conditions known to be important for audio-visual cuing becoming stronger than unimodal ones are that the (1) auditory and visual stimuli should be presented from more-or-less the same direction (Ho et al., 2007, 2009) and that (2) a high perceptual load should be used (Spence and Santangelo, 2009; Spence, 2010). In the current experiment, visual and auditory stimuli were presented from roughly the same spatial direction (at least on the horizontal plane; when the visual stimuli were on screen). The perceptual load of the procedure was rather high inasmuch as the participants also performed a secondary task. Therefore, the failure to find any benefit of congruent audio-visual stimulation over incongruent stimulation in veridical perception may rather be explained by attentional effects (see below) than by the absence of cross-modal integration. However, as the main screening procedure did not directly test audiovisual integration (only asking about the subjective experience of binding together the sounds and the visual objects), this possibility cannot be completely ruled out. Evidence suggesting that the audiovisual binding could be attention-dependent was obtained by Van Ee et al. (2009). In our experiments, one possibility is that in the *Incongruent* conditions, the participants' attention was drawn to the sounds rather than to the visual objects. This would explain why the influence of spatial separation of the sound sources on stream segregation was stronger with incongruent than with congruent visual cues. That is, the congruency between auditory and visual stimuli could take away attentional resources from evaluating spatial separation as a cue for auditory stream segregation. However, the performance in the visual task, which required participants to focus their attention on the visual objects, was not affected by audio-visual congruency. This implies that attentional effects cannot fully explain the absence of the benefit of congruent over incongruent audio-visual stimulation.

Alternatively, it is possible that the processes of auditory stream segregation do not utilize visual information. Existing data suggest that sensory integration is likely occurring at multiple processing stages (Kayser et al., 2012). Early interactions between auditory and visual modalities have been demonstrated in a number of studies (Giard and Peronnet, 1999; Fort et al., 2002; Besle et al., 2005; Cook and Van Valkenburg, 2009). Furthermore, visual effects on auditory object formation have been demonstrated in a study utilizing a bistable auditory perceptual phenomenon similar to the one employed in the current study (Rahne et al., 2007). Rahne et al. (2007) found that perceptual organization of the ambiguous auditory input was significantly influenced by synchronized presentation of visual stimuli compatible either with the within-stream (segregated) or across-stream (integrated) sound pattern. The mismatch negativity component (MMN) was elicited only when the visual input biased the sounds toward the two-stream organization, suggesting that the underlying audio-visual interaction occurred prior to the formation of the memory representation involved in the MMN deviance detection process. Similar effects have been obtained for a perceptually stable sound sequence (Rahne and Böckmann-Barthel, 2009). However in these studies only non-spatial audio-visual cues were employed. Therefore, it is possible that in contrast to temporal audio-visual congruency, spatial congruency exerts no or only weak influence on the auditory perceptual organization. In support of this conclusion, some results suggest that separate competing object representations may exist at multiple levels of processing (e.g., Blake and Logothetis, 2002; Tong et al., 2006). The question of whether audio-visual bistable perception is mediated by distributed intramodal mechanisms or is governed by a supramodal central mechanism was tested by Hupé et al. (2008) using sounds evoking auditory streaming and either visual plaids or visual stimuli evoking apparent motion. The auditory and visual stimuli had weak or strong cross-modal congruency, the latter being achieved by introducing spatial and temporal coincidence between the two modalities. The visual stimuli had no influence on the auditory switching statistics, indicating that bistability can co-occur independently in different sensory modalities. This interpretation is supported by the lack of a congruency effect on performance in the present visual task. Further, we have already noted above that the interaction between the effects of cross-modal congruency and spatial separation on sound organization probably stems from attentional differences between the congruent and incongruent conditions. Thus, the current results support models of modality-specific competition for perceptual decision and awareness.

One may wonder why the lack of advantage of congruent over incongruent audio-visual stimulation in auditory stream segregation does not apparently affect veridical perception in everyday situations. However, multistable auditory stimulus configurations (i.e., stimuli that are deprived of disambiguating cues) are very rare in everyday environments. Thus in most cases, visual information is not required to decide between alternative sound organizations. Therefore it may not have been sufficiently advantageous for the brain to utilize visual information for this purpose. This is not the case for example in identifying speech sounds, and indeed there are several results showing that e.g., lip movements affect the identification of speech sounds (see e.g., the McGurk effect; McGurk and MacDonald, 1976). Note that the previous suggestion that audio-visual integration follows auditory stream segregation does not mean that audio-visual integration does not take place at all. Further, when it comes to localizing objects, the dominance of visual over auditory information is obvious in most cases (see e.g., the ventriloquist effect; Howard and Templeton, 1966; for a review, cf. Recanzone, 2009). Thus in everyday life, we are not especially sensitive to spatial incongruence between auditory and visual stimuli. This leads us to suggest that the task requiring participants to continuously integrate between the auditory and visual objects took up more attentional resources than when they realized that the two were fully incongruent and concentrated primarily on the sounds.

We found a clear effect of sound source spatial separation on perceptual organization, which is consistent with the results of our earlier unimodal study (Bőhm et al., 2013) that also used moving sounds. It is also consistent with previous studies testing the effects of spatial separation between sound sources on auditory stream segregation (Cusack et al., 2004; Denham et al., 2010; Szalárdy et al., 2013). Sounds originating from different directions are more likely to be perceived as separate objects than sounds emitted from roughly the same direction.

Bőhm et al. (2013) found that auditory motion decreased the average perceptual phase durations of both the “integrated” and the “segregated” percepts compared to stationary sound sources without affecting the balance between the proportions of these perceptual organizations. The current results corroborated these findings: the mean durations of all percepts were lower for moving sound sources compared to stationary ones. Shorter average phase durations correspond to faster switching between the alternative sound organizations. Thus it appears that motion exerted a rather strong destabilizing effect on the perceptual organization. The increased rate of perceptual switching may be due to the auditory system continuously re-evaluating the spatial separation between the moving sound sources. The significant effect of motion obtained in the visual task could also indicate frequent re-evaluation of the stimulus position: listeners were less accurate in the *Moving* compared with the *Stationary* conditions. Besides destabilizing, motion had no further effect on auditory perceptual organization. In that regard, the current results support the main conclusion of Bőhm et al. (2013) who suggested that cues based on the Gestalt notion of common fate, at least those stemming from moving sound sources, are not effective in auditory stream segregation.

### Conflict of interest statement

The authors declare that the research was conducted in the absence of any commercial or financial relationships that could be construed as a potential conflict of interest.
